# Impact of Empire Expansion on Household Diet: The Inka in Northern Chile's Atacama Desert

**DOI:** 10.1371/journal.pone.0008069

**Published:** 2009-11-26

**Authors:** Sheila Dorsey Vinton, Linda Perry, Karl J. Reinhard, Calogero M. Santoro, Isabel Teixeira-Santos

**Affiliations:** 1 Department of Anthropology, University of Kentucky, Lexington, Kentucky, United States of America; 2 Archaeobiology Program, Department of Anthropology, Smithsonian National Museum of Natural History, Washington D.C., United States of America; 3 School of Natural Resources, University of Nebraska – Lincoln, Lincoln, Nebraska, United States of America; 4 Instituto Alta Investigación, Departamento de Antropología, Universidad de Tarapacá, Arica, Chile; 5 Centro de Investigaciones del Hombre en el Desierto, Arica, Chile; 6 Escola Nacional de Saúde Pública, Fundação Oswaldo Cruz, Rio de Janeiro, Brazil; University of Cape Town, South Africa

## Abstract

The impact of expanding civilization on the health of American indigenous societies has long been studied. Most studies have focused on infections and malnutrition that occurred when less complex societies were incorporated into more complex civilizations. The details of dietary change, however, have rarely been explored. Using the analysis of starch residues recovered from coprolites, here we evaluate the dietary adaptations of indigenous farmers in northern Chile's Atacama Desert during the time that the Inka Empire incorporated these communities into their economic system. This system has been described as “complementarity” because it involves interaction and trade in goods produced at different Andean elevations. We find that as local farming societies adapted to this new asymmetric system, a portion of their labor had to be given up to the Inka elite through a corvée tax system for maize production. In return, the Inka system of complementarity introduced previously rare foods from the Andean highlands into local economies. These changes caused a disruption of traditional communities as they instituted a state-level economic system on local farmers. Combined with previously published infection information for the same populations under Inka rule, the data suggest that there may have been a dual health impact from disruption of nutrition and introduction of crowd disease.

## Introduction

The influence of state-level civilizations on indigenous societies has long been studied, especially from the perspective of the emergence of infections disease and malnutrition. Sensational discoveries of ancient DNA in human coprolites (desiccated feces) have highlighted the importance of coprolites in the study of these social interactions in the Americas [1], [2]. The analysis of series of coprolites can be used to evaluate hypotheses regarding changes of diet and parasitism between time periods and cultures using long-standing theories and methods [3], [4]. We undertook a study of coprolites from pre-Inka and Inka period sites to evaluate the impact of the state on local health and diet. The sites are located in the Lluta Valley near Arica, Chile in the northern Atacama Desert. Previously, we found that crowd parasites (*Enterobius vermicularis* or pinworm) emerged when the indigenous farmers fell under Inka control [5]. Fish tapeworm, *Diphyllobothrium pacificum*, was present in Inka times but was not observed in pre-Inka contexts. Our next question related to the impact of the Inka on local diet. To answer this we developed a new method for analyzing starch from coprolites.

Dietary change relates to how local communities become integrated into the political, economic, and ideological systems imposed by the state. This process of integration is the focus of many studies of ancient empires. Such studies are concerned with details such as how indigenous communities negotiated new political circumstances, to what extent they lost their traditional pattern of life, and how much of their own economic sustainability was jeopardized or changed as a consequence of state demands [6]. Here we show how the influence of the Inka Empire changed local diet through analysis of a series of human coprolites from two pre-Hispanic periods in the Lluta River Valley. We focus on starch analysis to evaluate the diversity and preparation of Andean grains and tubers. The utility of starch analysis in paleoethnobotanical studies has been demonstrated from more than a decade [7]. This approach has rarely been applied or published from coprolite analysis.

In the South Central Andes, with a pivotal center in the Lake Titicaca basin, the Inka took advantage of previous Andean economic strategies for accessing and circulating products among a diverse range of ethnic groups situated in a highly variable Andean ecology. Murra described the redistribution principle as “ecological complementarity” that he originally defined as vertical archipelago or “verticality.” In this system, distinct altitudinal Andean ecological tiers were linked through direct control by each political ethnic group [8], [9]. According to his model, highland people maintained colonies in the lowlands on both the Pacific and Amazonian sides of the Andes. This organizational strategy affected distribution of food, prestige, and ceremonial goods. Early colonial written records state that prior to the Spanish conquest, highland Aymara-speaking groups (Lupaqa, Pacaje, and later in colonial times the Caranga) maintained colonies on productive enclaves on the coastal valleys of Arica, Chile [9], [10], [11]. This region includes our study area, the Lluta River Valley near Arica, Chile [12]. Using coprolite analysis, we are testing the hypothesis that the Inka system introduced highland Andean crops into the Lluta Valley while reducing local consumption of taxable maize (*Zea mays*).

We analyzed a series of 48 coprolites from Lluta Valley archaeological sites to test the hypothesis that the Inka Empire introduced a different framework for crop production, circulation, and consumption that reached domestic levels of local communities. This pattern has been described previously in the central Andes of Peru [13]. The coprolite samples were obtained from dry sieving archaeological deposits excavated from cane and reed wall domestic structures of six hamlets in the coastal section of the Lluta Valley, 20 to 25 km from the coast [14]. These sites show evidence of occupation during the Inka Late Period (AD 1400–1532), and the pre-Inka Late Intermediate Period (AD 1100 –1400). The coprolites were recovered from 21 contexts at six different sites. The sites show no evidence of Spanish colonial activities.

Coprolites are common in Atacama Desert archaeological sites [15], [16], [17]. Although coprolites have been used to describe dietary development over long periods of time, only rarely are coprolites used to test specific hypotheses regarding culture change. Starchy foods were a prominent portion of the Andean diet and, therefore, the remains of these plants are abundant in coprolites. Due to several biases in the history of the development of the discipline, starch analysis as a source of dietary data has lagged in coprolite studies [18], [19], [20].

## Results and Discussion

It is noteworthy that for the Lluta Valley coprolites, all but two contained starch. The near ubiquity of starch granules in the coprolites demonstrates that starch is the most productive area for diet reconstruction and comparison in the Atacama region as recently recommended by reviewers of Moche coprolite analysis [19].

The 25 pre-Inka coprolites showed a high consumption of maize starch and limited consumption of tubers [20]. Only two samples contained anything other than maize starch. One contained maize and *yuca* (*Manihot esculenta*) and another contained maize and *oca* (*Oxalis tuberosa*). In the 23 Inka period coprolites, a greater presence of tubers was represented. Six contained *yuca* starch. Four contained *oca* starch. Four contained potato (*Solanum* spp.) starch. Six contain unknown starch and 21 contained maize starch. Therefore, in addition to maize, three or more types of tubers or starchy plants were eaten.

Insignificant chi square values for individual starch comparisons were obtained for maize (0.185), *yuca* (1.169), *oca* (0.216), and potato (1.361). However, when all tubers were combined a significant chi square value of 7.312 was obtained.

A subset of the coprolite series was analyzed for concentration values following a method of pollen analysis recently adapted to coprolite analysis [21]. The maize counts are presented in Table 1 with calculated concentrations of starch granules in terms of granules per gram of coprolite. In general, there are more maize starch granules in pre-Inka coprolites. Most importantly, the numbers of damaged starch granules are higher in pre-Inka times. The average ratio of damaged starch to pristine starch granules for the Pre-Inka is 9.6. The ratio for the Inka is 1.8.

**Table 1:** Data from two coprolite analysis.

Sample	Maize	*Yuca*	*Chuñ'u*	*Oca*	Pristine Maize Count	Altered Maize Count	Total Maize Concentration starch/g	Ratio of Altered Maize to pristine maize
INKA								
1	X		X		24	15	4,238	0.625
2	X				33	13	3,189	0.39
3	X	X	X		95	99	274,025	1.04
4	X				14	26	15,586	1.86
5	X				9	2	15,537	0.22
6	X	X			30	79	419,567	2.63
7	X				---	---	---	---
8	X				52	41	15,013	0.79
10	X		X	X	0	1	159	1.0
11	X				6	42	5,370	7.0
16	X	X			---	---	---	---
17	X	X			---	---	---	---
19	X				63	159	627,150	2.52
25					---	---	---	---
26	X				---	---	---	---
28	X				---	---	---	---
29	X				---	---	---	---
30					---	---	---	---
31	X				---	---	---	---
37	X	X	X	X	---	---	---	---
65	X				---	---	---	---
67	X			X	---	---	---	---
70	X	X		X	---	---	---	---
Total or Mean	21	6	4	4	Mean = 32.6	Mean = 47.7	Mean = 137,983	Mean = 1.8
PRE-INKA								
59	X				128	872	11,300,000	6.81
60	X	X			---	---	---	---
61	X				15	201	1,220,400	13.4
71	X				25	143	111,671	5.72
72	X			X	52	7	4,358	0.13
73	X				33	48	9,737	1.45
76	X				7	49	13,678	7.0
77	X				---	---	---	---
78	X				---	---	---	---
79	X				8	49	5,551	6.13
80	X				---	---	---	---
82	X				14	85	24,860	6.07
84	X				2	31	10,654	15.5
85	X				2	62	22,600	31.0
86	X				12	144	29,380	12.0
Total or Mean	15	1	0	1	Mean = 27.1	Mean = 153.7	Mean = 1,159,353	Mean = 9.56

The first analysis assessed the presence/absence of plant taxa. The second analysis measured the pollen concentration of a subset of samples analyzed originally. The first columns show the presence/absence results from Vinton's analysis of starch diversity for maize (*Zea mays*), *yuca* (*Manihot esculenta*), *oca* (*Oxalis crenata*), *chuñ'u* (*Solanum tuberosum*) 17. The last 4 columns present the data from 200 starch grains counts focusing on maize results only. Other starch types including *yuca*, maize, quinoa, *oca* and unknown types were encountered but are not presented in this table. Concentration refers to the calculated numbers of starch granules per gram of coprolite. Pristine maize refers to starch granules that show now evidence of preparation by fermentation or cooking. Altered maize refers to starch granules that show erosion of the internal or external surface consistent with effect of fermentation or cooking.

**Figure 1:**
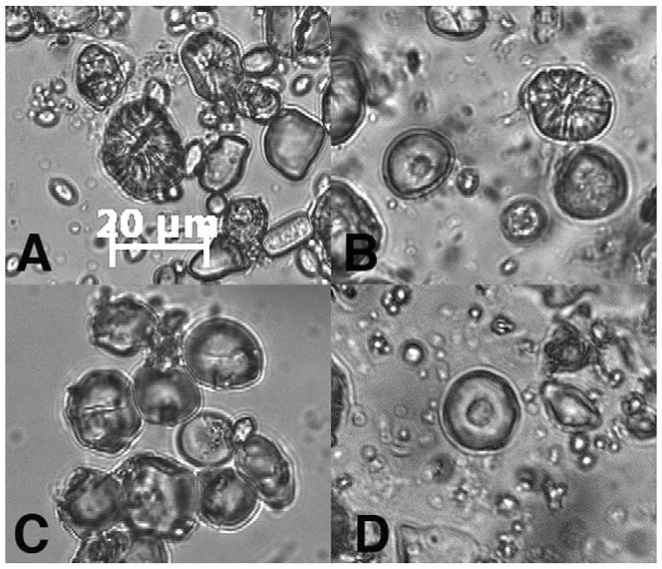
Comparison of ancient starch with modern starch from experimental preparation. All images are of equivalent scale. A. Maize starch granules from the experimental fermentation experiment showing furrowing damage. B. Maize starches derived from archaeological sample #51 showing furrowing damage from fermentation and scooping from grinding. C. Unaltered maize meal prior to the experiment. D. Scooping of maize starch produced in a grinding experiment.

A common maize-based food in the Andes was chicha. Chicha is a drink made of fermented maize. Our data from experiments in brewing maize meal into chicha demonstrate that the damaged grains are evidence of the consumption of this fermented beverage. When examined microscopically, the damaged starch granules displayed surface furrowing that is identical to many of the maize granules recovered from the coprolite samples (Fig. 1). Additionally, the archaeological samples are characterized by damage that has been produced by the grinding of uncooked, dried maize in laboratory settings (Fig. 1). Absent in the coprolite samples examined by coauthor LP were gelatinized starches, those that have been damaged by heat typical of cooking processes.

When the coprolite samples are viewed in light of the experimental results, the following scenario can be reconstructed. The archaeological starches have been subject to grinding and fermentation, but not to cooking. Modern chicha can be prepared either raw or cooked, with the heated chicha having a much longer stable shelf life than the uncooked. Thus, this chicha was made from ground, dried maize, and was expected to be drunk within a short period of time. The coprolite data show that pre-Inka Empire farmers consumed more of this maize chicha than farmers who fell under Inka rule.

The ethnobotanical data demonstrate that under Inka management there was an increase of consumption of starchy root crops (*oca*, *yuca* and *chuñ'u* [dehydrated potato]) [20]. *Oca* and *chuñ'u* could not have been cultivated locally, and, thus would have been brought into the sites from the highlands [14], [20]. Simultaneously, there was a reduction in the consumption of maize in the form of chicha. This decreased production was not recognizable in assemblages of artifacts [14]. In contrast, at the Sausa site in Central highland Peru, there is a generalized increase in maize production and consumption along with a reduction in the consumption of tubers under the Inka regime [13], [22].

Based on the maize concentration data of processed versus unprocessed starch granules, we can interpret that maize has a double function of food and ceremony. Chicha was produced in the Lluta valley under the *corvée* labor Inka state tax system known as *mit'a* [23], [24], [25], [26]. Contrary to the pre-Inka period, a small part of maize was consumed by as chicha by local commoners, while the main bulk was incorporated into the state circulation system, to be consumed elsewhere. Therefore, the relative lack of altered maize starch in Inka period coprolites indicates that only a portion of processed maize in the form of fermented maize chicha was distributed back to the village populations while the rest was taken by the state in the *mit'a* and the redistrubution Inka system. This pattern has been previously hypothesized by Morris and Thompson and Murra [27], [28]. Another important change in the domestic economy was the introduction of tuber crops (potatoes and oca) from the highlands. These crops circulated alongside other exotic goods found both at domestic and funerary contexts in the Lluta valley [12], [14].

For more than a decade, the value of starch residue analysis has been demonstrated in archaeological contexts [29], [30]. The results from this study establish that starch from coprolites can both reveal details of plant production in state economic systems and support previous hypotheses [25], [26]. The pre-Inka Lluta Valley traditional life focused on local production and consumption of chicha. This tradition was largely lost under the Inka. The data show that the farmers of the Lluta Valley negotiated the Inka political circumstances by consuming less, and perhaps producing more, of their traditional maize chicha. This reduction was a consequence of state demands for corn to transform it into chicha in the Inka tax labor and distributional system. The fact that they became absorbed in the Inka vertical redistribution system is represented by the larger presence of exotic tubers in Inka times. This change may represent the fact that the farmers' sustainability was altered by the Inka expansion.

In summary, the inclusion of the farming society of the Lluta Valley in the Inka Empire resulted in dietary changes. Previously, we found a difference in infectious disease[5]. There is a connection between the two relating to the expansion of the Inka State in the Lluta Valley. Crowd pathology, such as pinworm infection, increased in the Lluta valley when the Inka established large village communities that replaced earlier dispersed small farms. At the same time, the subsistence was altered. Maize chicha consumption declined as highland Andean tubers were introduced into the communities. Further study of Lluta Valley mummies and skeletons must be done to assess the levels of pathology that are associated with parasitological and dietary developments of the Inka Empire.

## Materials and Methods

The methods used for processing the coprolites involved rehydration, disaggregation, screening, and microscopic analysis [5], [19], [20]. One *Lycopodium* spore tablet, containing 11,300 plus or minus 400 spores, was added to each gram of sample. The addition of a known number of identifiable spores enables an estimation of pollen grains, starch grains, and parasites per gram of coprolite [21]. The samples were then immersed in 0.5% NaPO_3_ in 250 ml beakers. The samples were allowed to rehydrate for 48 hours. They were stirred with a magnetic stir bar to disaggregate the components. The disaggregated residues were washed through a 0.25-mm mesh screen to separate large components from microscopic components including starch. The sediment left on the screens was dried and analyzed. The microscopic residues were then concentrated by centrifugation. The microscopic residues were placed into 4 dr vials with 95% ethanol. Three microscope slides were made from each sample of microresidues from the 48 coprolites. The starch grains were analyzed with a Jenaval research microscope using polarized light.

Starch can easily be identified microscopically with a polarizing light. The granules fluoresce in a pinwheel configuration called interference crosses. Polarization can be very brilliant to very weak [31]. Starch can be identified to family and genus level by size, shape, and sometimes fissures present in the granule. To evaluate the amount of starch to fiber for several Andean foods, samples from various plants that grow in the Andes highlands or that comprised a large portion of the diet were collected and prepared into a starch reference collection. The collection included maize (*Zea mays*), *yuca* (*Manihot esculenta*), *oca* (*Oxalis crenata*), *chuñ'u* (*Solanum* spp.), *maca* (*Lepidium meyenii*), *arracacha* (*Arracacia xanthorrhiza*) and *quinoa* (*Chenopodium quinoa*).

Later, a subset of 11 Inka coprolites and 10 pre-Inka coprolites were analyzed for maize starch concentrations. Specifically, the numbers of pristine maize starch granules and altered starch granules were counted and the concentrations of these were calculated by use of microfossil concentration technique [21]. Experiments were completed with modern maize to replicate the alteration observed in the prehistoric starch. During the examination of archaeobotanical samples that include starch granules, it is not unusual to observe morphological features of these residues that appear to have been altered from their native states. In attempts to understand these morphological changes, several experimental studies have been undertaken. These studies have replicated many processes believed to be typical in the ancient preparation of plant foods and include the grinding of seeds with stone tools, freeze drying of potatoes, cooking of various foods with water, and fermentation of grain with yeast [32], [33], [34]. The experimental procedures resulted in starch granules with clearly defined patterns of damage that are diagnostic of the types of processing. Thus, with careful analysis, archaeological starch granules that bear damage identical to that achieved through modern experimental processing can be reliable indicators of the use of these processes in the past [32].

For the purposes of replicating damage caused by fermentation during the production of chicha, a commercially prepared corn meal was placed in a container with filtered water and human saliva. This mixture was placed in a closed but unsealed plastic container, and was allowed to ferment outdoors for four days during which time ambient temperatures ranged between 21 and 33 degrees Centigrade. At the end of this time, the mixture smelled strongly like sourdough bread starter, an indication that the fermentation had been successful. To determine the type of damage caused by the process, starch grains from the fermented mixture were examined microscopically (Fig. 1).
